# Arrhythmic substrate, slowed propagation and increased dispersion in conduction direction in the right ventricular outflow tract of murine *Scn5a+/*− hearts

**DOI:** 10.1111/apha.12324

**Published:** 2014-07-09

**Authors:** Y Zhang, L Guzadhur, K Jeevaratnam, S C Salvage, G D K Matthews, W J Lammers, M Lei, C L-H Huang, J A Fraser

**Affiliations:** 1 Physiological Laboratory, University of CambridgeCambridge, UK; 2 Heart Centre, Northwest Women's and Children's HospitalXi'an, China; 3 Perdana University-Royal College of Surgeons IrelandSelangor, Malaysia; 4 Department of Physiology, College of Medicine and Health SciencesAl Ain, UAE; 5 Department of Pharmacology, University of OxfordOxford, UK; 6 Department of Biochemistry, University of CambridgeCambridge, UK

**Keywords:** Brugada Syndrome, conduction velocity, Na^+^ channel, right ventricular outflow tract

## Abstract

**Aim:**

To test a hypothesis attributing arrhythmia in Brugada Syndrome to right ventricular (RV) outflow tract (RVOT) conduction abnormalities arising from Na_v_1.5 insufficiency and fibrotic change.

**Methods:**

Arrhythmic properties of Langendorff-perfused *Scn5a*+/− and wild-type mouse hearts were correlated with ventricular effective refractory periods (VERPs), multi-electrode array (MEA) measurements of action potential (AP) conduction velocities and dispersions in conduction direction (CD), Na_v_1.5 expression levels, and fibrotic change, as measured at the RVOT and RV. Two-way anova was used to test for both independent and interacting effects of anatomical region and genotype on these parameters.

**Results:**

*Scn5a+/*− hearts showed greater arrhythmic frequencies during programmed electrical stimulation at the RVOT but not the RV. The *Scn5a*+/− genotype caused an independent increase of VERP regardless of whether the recording site was the RVOT or RV. Effective AP conduction velocities (CV†s), derived from fitting regression planes to arrays of observed local activation times were reduced in *Scn5a*+/− hearts and at the RVOT independently. AP conduction velocity magnitudes derived by averaging MEA results from local vector analyses, CV*, were reduced by the *Scn5a*+/− genotype alone. In contrast, dispersions in conduction direction, were greater in the RVOT than the RV, when the atrioventricular node was used as the pacing site. The observed reductions in Na_v_1.5 expression were attributable to *Scn5a*+/−, whereas increased levels of fibrosis were associated with the RVOT.

**Conclusions:**

The *Scn5a*+/− RVOT recapitulates clinical findings of increased arrhythmogenicity through reduced CV† reflecting reduced CV* attributable to reduced Na_v_1.5 expression and increased CD attributable to fibrosis.

The Brugada Syndrome (BrS) is characterized by an increased risk of arrhythmogenic sudden cardiac death particularly in middle aged (approx. 40–45 years) males (Brugada *et al*. 2002, Priori *et al*. 2002, Antzelevitch *et al*. 2005, Eckardt *et al*. 2005, Probst *et al*. 2010). It has been thought to be primarily a right ventricular (RV) disease (Antzelevitch *et al*. 2005). It is often associated with electrocardiographical (ECG), right praecordial ST segment elevation (Brugada & Brugada 1992, Brugada *et al*. 1998), right bundle branch block and ventricular tachycardia (VT) or fibrillation (VF) originating from the RV outflow tract (RVOT) (Nagase *et al*. 2002, Haissaguerre *et al*. 2003, Kofune *et al*. 2011). However, the detailed pathophysiology producing re-entrant waves of excitation remains uncertain. A *repolarization disorder* was suggested on the basis of the reduction in Na^+^ current (*I*_Na_) in relationship to transient outward K^+^ currents (*I*_to_). This would shorten RV epicardial action potential durations (APD) and thereby produce a transmural dispersion of RV action potential waveform (Kurita *et al*. 2002, Antzelevitch *et al*. 2007). Alternatively, reduced *I*_Na_ would be expected to slow action potential (AP) conduction particularly in the RVOT (Nagase *et al*. 2002, Lambiase *et al*. 2009) assuming that its slowly conducting embryonic phenotype persists into the adult (Boukens *et al*. 2013). The RVOT has thus been implicated in both the electrocardiographical abnormalities and the delayed epicardial conduction associated with potentially fatal ventricular arrhythmia in BrS (Berruezo *et al*. 2004, Morita *et al*. 2008) in a *depolarization disorder* hypothesis (Kasanuki *et al*. 1997, Tukkie *et al*. 2004, Coronel *et al*. 2005, Meregalli *et al*. 2005, Postema *et al*. 2008).

Ventricular fibrillation in patients with BrS can often be induced at the RVOT (Morita *et al*. 2003). This might relate to structural abnormalities in the RVOT in BrS, detectable as wall motion abnormalities (Takagi *et al*. 2001) and fibrotic or fatty endocardial infiltration (Papavassiliu *et al*. 2004). Some clinical studies have also explored for electrophysiological abnormalities localized to the RVOT. However, such studies in human subjects were confined to non-invasive assessments of RVOT conduction delays employing body surface mapping (Izumida *et al*. 2004), signal-averaged ECGs (Hisamatsu *et al*. 2004) or tissue Doppler echocardiography (Tukkie *et al*. 2004). Nevertheless, there have been a limited number of invasive human studies. These demonstrated endocardial RVOT activation delays (Kanda *et al*. 2002) as well as electrogram prolongation and steeper activation recovery intervals (ARI) and restitution curves in RVOT endocardial recordings (Lambiase *et al*. 2009).

However, clinical studies investigating epicardial heterogeneities in depolarization and repolarization are difficult to perform. One report described epicardial electrograms from the conus branch of the right coronary artery running over the RVOT surface. This demonstrated activation delays not found in the endocardium (Nagase *et al*. 2002), as well as a shortening of ARIs in the RVOT using an epicardial catheter in the great cardiac vein during ST elevation. Another study demonstrated epicardial, but not endocardial spike-and-dome AP waveforms during open chest surgery (Kurita *et al*. 2002). An explanted BrS heart in which VF was induced by programmed stimulation, showed a subendocardial rather than subepicardial re-entrant activation, with RV activation slowing in an absence of transmural repolarization gradients in the RVOT. Its RV contained abundant fibrous and adipose tissue (Coronel *et al*. 2005). A further explanted BrS heart combined ST segment elevation with a reduced local activation in basal RV subepicardium. This showed a fibrosis and fatty infiltration that in combination with altered *I*_Na_, and consequently *I*_CaL_ and *I*_to_, could produce a current-to-load mismatch at its junction with more normal myocardial tissue. This could account for both reduced conduction velocities and the characteristic BrS ECG pattern (Hoogendijk *et al*. 2010a,b). Such a suggestion was compatible with results from experimental and modelling studies (Hoogendijk *et al*. 2011).

Of experimental models for BrS, the RV pharmacological wedge preparation precludes localization of arrhythmias in the RVOT. However, an *in vivo*, closed chest canine study demonstrated ECG changes characteristic of BrS following cooling of a small epicardial RVOT region, and produced a ‘spike and dome’ appearance in the monophasic AP (MAP) in the epicardium. However, this model did not reproduce the loss of the AP dome (Nishida *et al*. 2004). Experimental systems containing genetic modifications directly replicating those known to exist in BrS may provide more specific models for the associated physiological abnormalities whilst permitting studies impractical in human subjects. Up to 30% of BrS patients have *SCN5A* mutations involving the cardiac voltage-gated Na^+^ channel *α*-subunit (Na_v_1.5) (Chen *et al*. 1998, Antzelevitch *et al*. 2007, London *et al*. 2007). A murine, Na_v_1.5 haploinsufficient, heterozygotic, *Scn5a+/*−*,* model (Papadatos *et al*. 2002) has proven useful in physiological studies of both atrial and ventricular arrhythmic properties in BrS (Lei *et al*. 2005, Stokoe *et al*. 2007, Hao *et al*. 2011). *Scn5a+/*− hearts show a 50% reduction in RV Na_v_1.5 expression and in *I*_Na_ (Martin *et al*. 2012). Loss of function *SCN5A* mutations are associated with a complex range of phenotypes that include sinus node dysfunction and progressive conduction disorders in both clinical situations (Wilde & Coronel 2008) and in the mouse model (Papadatos *et al*. 2002). The *Scn5a*+/− mouse reproduces many key features of human BrS. It shows ECG ST elevation (Martin *et al*. 2010b) and ventricular arrhythmogenesis accentuated by flecainide and relieved by quinidine (Martin *et al*. 2010a,b), particularly in the right ventricular (RV) epicardium (Matthews *et al*. 2013) with delayed RV response latencies and increased transmural repolarization gradients (Martin *et al*. 2010b). It also shows an age-dependent RV fibrosis (van Veen *et al*. 2005, Jeevaratnam *et al*. 2010) in line with clinical findings (Coronel *et al*. 2005).

The present study first explored whether murine *Scn5a+/*− hearts replicated the arrhythmic properties of RV and RVOT sites observed in human BrS under conditions of extrasystolic intraluminal stimulation. The experiments then related these arrhythmic properties to differences between their electrophysiological features and those of WT hearts. These included ventricular effective refractory periods (VERPs), conduction velocities and dispersions in conduction direction (CD). These physiological findings were next compared with differences in Na_v_1.5 expression and tissue fibrosis. Differences in all these parameters were then statistically correlated with either the genotype or the site at which the parameter was measured.

## Materials and methods

All procedures were performed in conformity with good scientific practice recommendations (Persson 2013, Zhang *et al*. 2013). The studies described here were based on a total of 23 WT and 21 *Scn5a+/*− hearts from inbred 129/sv mice aged 3–8 months.

### Arrhythmogenic properties of isolated Langendorff-perfused hearts

**Figure 1:**
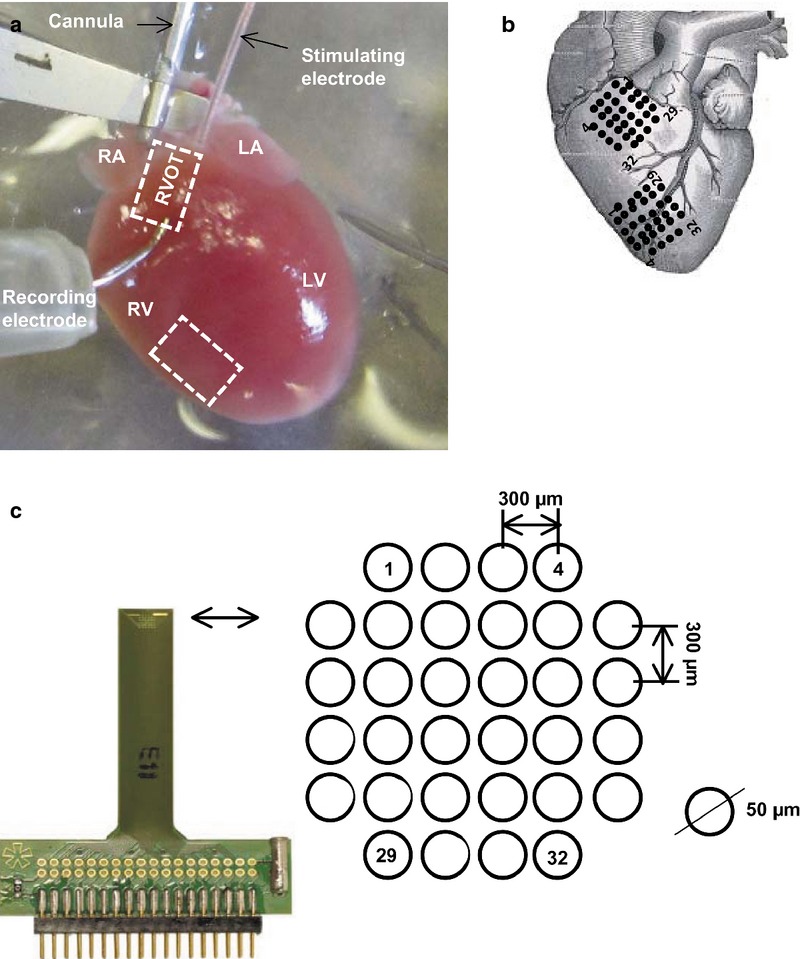
Electrophysiological experimental set-up for multi-array recordings from the right ventricular outflow tract and right ventricle. (a). Langendorff-perfusion set-up. (b) Placing positions and recording orientations on the free walls of the RVOT and RV for the multi-electrode array (MEA). (c) MEA configuration consisting of 32 electrodes in a square 1.5 × 1.5 mm matrix, with an electrode diameter 50 *μ*m, inter-electrode distance 300 *μ*m. LA, left atrium; RA, right atrium; LV, left ventricle; RV, right ventricle; RVOT, right ventricular outflow tract.

The electrophysiological experiments employed isolated Langendorff-perfused hearts, prepared and studied under conditions and solutions described on earlier occasions (Matthews *et al*. 2013, Zhang *et al*. 2013). Hearts were first studied at their intrinsic rates without applied stimulation. Endocardial pacing was then delivered using a 1-Fr bipolar mouse pacing catheter (NuMED; Hopkinton, NY) inserted into the RV through the pulmonary artery. This delivered stimulation first to the endocardium of the RVOT free wall <2 mm below the pulmonary artery, and then as close to the RV apex as could be reached by the catheter (Fig. 1a). This stimulation order was reversed in half the hearts studied. First, regular, 8 Hz, pacing stimuli used 2-ms square-wave pulses whose amplitude was adjusted to twice the excitation threshold (DS2A isolated constant voltage stimulator; Digitimer, Welwyn Garden City, UK). This was followed by a programmed electrical stimulation (PES) procedure. This began by imposing standard 8 Hz baseline-pacing stimuli for 20 s. The subsequent drive trains consisted of cycles of eight paced beats (S1) each followed by an extrastimulus (S2) initially imposed at an S1–S2 interval equal to the pacing interval, then reduced by 1 ms with each subsequent cycle until the VERP was reached.

Our strategy of endocardial stimulation permitted measurement of electrical signals using an epicardial monophasic action potential (MAP) recording electrode (Hugo Sachs, Harvard Apparatus, Edenbridge, Kent, UK) successively placed against the RVOT and the RV free wall. The MAPs were pre-amplified with a NL100AK head stage, band-pass filtered (0.5 Hz–1 kHz: Neurolog NL 125/6 filter; Digitimer, Welwyn Garden City, Hertfordshire, UK) and digitized at a sampling frequency of 5 kHz with a micro 1401plus MKII laboratory interface (Cambridge Electronic Design, Cambridge, UK). Analysis of MAP waveforms was performed using Spike II software (Cambridge Electronic Design).

### Multi-electrode array recordings and quantification of effective epicardial conduction properties

WT and *Scn5a+/*− hearts (Fig. 1a,b) were subject to regular (8 Hz) pacing applied at (a) the endocardium using the 1-Fr bipolar pacing catheter inserted through a left atrial incision to access the atrioventricular node (AVN) and at (b, c) the epicardium of the LV (b) base and (c) apex using a custom-made suction electrode. Epicardial AP recordings were made using 32 channel MEAs (ME32-FAI-System; Scientifica, Uckfield, UK) applied to the RVOT and RV free walls simultaneously. Each MEA contained 32 electrodes of 50 *μ*m diameter in a 1.5 × 1.5 mm configuration with a 300-*μ*m inter-electrode distance (Fig. 1c), sampling at 10 kHz per channel. The potential at each recording site was recorded against a reference level provided by a single electrode placed remote from the recording array on the metal cannula through which the heart was perfused. This therefore provided monophasic recordings of the voltages generated at each recording site.

Previous studies have pointed out that the intrinsic deflection reflecting the AP upstroke corresponds to the negative going deflection in monophasic recordings of excitable activity (Dower 1962, Janse & Rosen 2006). Off-line determinations of local activation times (LATs) at each recording site were related to timings of the first detected waveform in the recording array at which the waveforms showed their maximal negative slopes. Such an approach agreed with determinations using their peak deflections.

These LATs were first used to derive effective epicardial conduction velocities (CV†), by fitting a regression plane to the LATs (King *et al*. 2013). The LATs were first visualized on three-dimensional plots to locate the largest area forming a uniform plane for optimization. Results were discarded if uniform areas covering at least 24 adjacent channels were not found: this would not be consistent with stable conduction velocities. However, very few (<5 of approx. 100) cardiac cycle recordings studied for each heart were thus excluded. In approximately half of the analyses, the entire array could be used in regression analysis. This gave multiple-*r* values between 0.90 and 0.98 corroborating the approximation procedure. The resulting CV† values, reflecting the reciprocal of the gradient of the best-fit plane, were expressed in dimensions of velocity (m s^−1^).

A local vector analysis then analysed the LATs in successive, neighbouring 2 by 2 square sets of four recording points successively labelled from L1 to L4 along the perimeter of each set. Row and column time difference vectors were then calculated from their corresponding LATs as 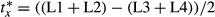 and 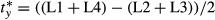 respectively. These in turn provided the magnitude, 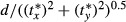 and direction, *θ*, of their contained velocity vector. A mean conduction velocity magnitude, CV*, could then be obtained by averaging the magnitude values from the entire array. The dispersion in CD, was obtained from the standard deviation of the corresponding *θ* values. Coherent conduction paths with low CD would give CV† values close to the corresponding CV*. Curved or incoherent conduction paths with high CDs would give CV† values less than the CV*.

### Western blot and histology

Age, litter and sex-matched hearts of further 6-month old WT and *Scn5a+/*− mice were excised for either Western blot or histological anatomical studies. Western blot analysis was used to estimate Na_v_1.5 protein expression in the RV and RVOT of both *Scn5a+/*− and WT hearts. Total protein was extracted from the RV and RVOT strip using lysis buffer containing (mm) 20 Tris–HCl (pH 8.0), 137 NaCl, 2 EDTA and 1% Triton x-100 and 10% glycerol. The protein concentration was measured using the Bio-Rad protein assay kit (Bio-Rad, Hemel Hempstead, Hertfordshire, UK). Bovine serum albumin was used as a standard, and all samples were normalized to the same concentration. Ten microgram of protein extract was separated by 4–8% SDS–PAGE gels, and transferred to polyvinylidene difluoride membranes (Immuno-blot PVDF Membrane; Bio-Rad). The membrane was blocked in 5% non-fat dried milk in PBST [phosphate-buffered saline (PBS), 0.05% (v/v) Tween 20 (Sigma-Aldrich, Gillingham, Dorset, UK), pH 7.4], and then probed with an anti-human Na_v_1.5 antibody, followed by a horseradish peroxidase-conjugated secondary antibody. The protein signal was visualized using the ECL Western blotting detection kit and Hyperfilm ECL (Amersham Biosciences, Bath, Avon, UK). The specimen was re-probed using an anti-GAPDH antibody (Chemicon, Watford, UK), which served as loading control. The anatomical studies used isolated hearts flushed with Krebs-Henseleit buffer (Matthews *et al*. 2013, Zhang *et al*. 2013) and perfused with 4% buffered formalin for 5 min before overnight formalin fixation. Gross transverse sections from apex to base then underwent routine tissue processing and paraffin embedding. The base and middle heart blocks were sectioned at a 7 *μ*m thickness towards the RVOT and RV for picrosirius red staining (Sigma-Aldrich), viewing, magnification and digital recording using the Nano Zoomer 2.0 Digital Pathology system (Hamamatsu, UK). Quantification of fibrosis was performed for each heart on four randomly selected photomicrographs representing sections spaced by a minimum distance of 14 *μ*m.

### Statistical analysis

Incidences of arrhythmia were analysed using Fisher's exact tests applicable to such categorical data. The remaining, continuous data, expressed as means ± SEM, were first tested using two-way anova for correlated samples analysis of variance (spss; IBM, Portsmouth, Hamps., UK and Graphpad Prism V6.1; La Jolla, CA, USA). Each two-way anova tested for effects of the categorical groups representing genotype (i.e. either WT or *Scn5a*+/−) or choice of recording site (i.e. either RV or RVOT) upon the continuous data obtained from the experimental measurements. Such measurements yielded values of VERP, CV†, CV* and CD, and characterized Na_v_1.5 expression and fibrotic change. This made it possible to demonstrate significant differences in these variates, and assess whether these differences could be attributed to independent or interacting effects of either genotype or anatomical site. Particular differences were then investigated by Bonferroni-corrected Student's unpaired *t*-tests to a significance level of *P* < 0.05.

## Results

### Programmed electrical stimulation demonstrates greatest arrhythmogenicity at the Scn5a+/− RVOT

**Figure 2:**
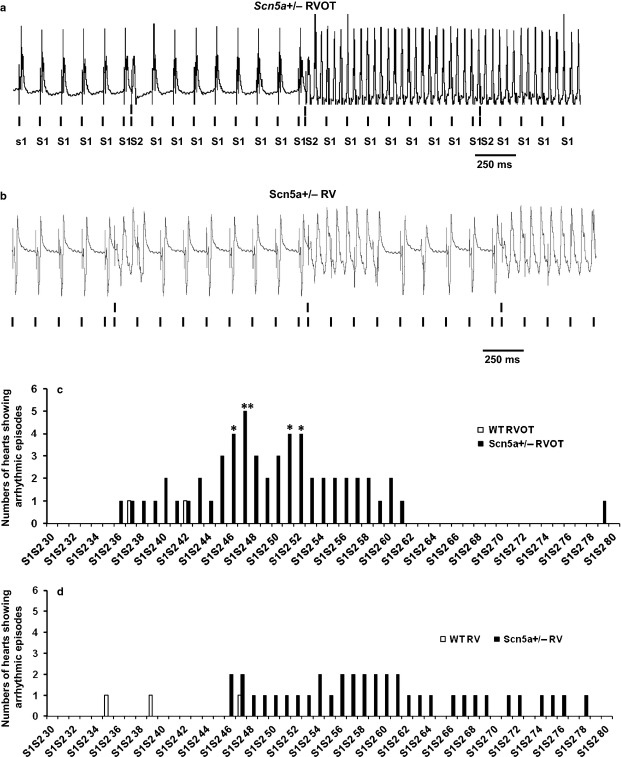
(a, b) VT episodes recorded from the free wall of the right ventricular outflow tract (RVOT) and right ventricle (RV) during programmed electrical stimulation (PES) of *Scn5a*+/− hearts. (a) sustained episodes of VT in the RVOT of a *Scn5a*+/− heart. (b) short episodes of VT in the RV of a *Scn5a*+/− heart. (c) and (d) summarize numbers of hearts showing arrhythmic episodes (vertical axis) at each progressively shortened S1S2 interval in the course of PES procedures (horizontal axis) applied to the *Scn5a*+/− (filled bar) and WT (open bar) during pacing at the RVOT (c) and RV (d). *P < 0.05; **P < 0.01.

The experiments first explored arrhythmic properties during intrinsic activity, regular endocardial pacing and PES. An occurrence of VT was defined as a period containing three or more consecutive ectopic beats detected by epicardial recording. Neither WT nor *Scn5a+/*− showed spontaneous arrhythmic activity during 15 min runs of intrinsic activity or 2 min runs of regular pacing. In contrast, application of two successive runs of PES provoked arrhythmia with greater frequency in *Scn5a+/*− compared to WT hearts. This took the form of either sustained (Fig. 2a) or short VT episodes (Fig. 2b). Categorical data comparing incidences of arrhythmia in *Scn5a*+/− and WT hearts with pacing at either the RVOT or RV was analysed by Fisher's exact test. With RVOT pacing, *Scn5a+/*− hearts showed greater arrhythmic incidences than WT: VT was then observed in 1 of 9 WT but 6 of 7 of *Scn5a+/*− hearts (*P* < 0.01). In contrast, with pacing at the remaining RV, WT and *Scn5a*+/− hearts showed indistinguishable incidences of VT (2 out of 7 vs. 4 of 6 hearts respectively: *P* > 0.05). Figure 2c,d plot the number of individual hearts (vertical axis) that showed arrhythmic incidents at particular S1S2 intervals (horizontal axis) during the PES protocol. They compare observations resulting from stimulation applied to the RVOT (c) and RV (d) of WT and *Scn5a*+/− hearts respectively. The asterisks indicate significant differences in the occurrence of arrhythmia between the *Scn5a*+/− and the WT. This representation demonstrated that more *Scn5a*+/− than WT hearts showed arrhythmia. For both RVOT and RV stimulation sites, when arrhythmia occurred, it appeared at longer S1S2 intervals in the *Scn5a*+/− than in the WT. Finally, with RV stimulation, fewer hearts showed arrhythmia. However, when arrhythmia did occur, it took place at longer S1S2 intervals than was the case for the RVOT. In conclusion, arrhythmic tendency was increased specifically with RVOT as opposed to RV stimulation in *Scn5a*+/− but not WT hearts.

### Ventricular effective refractory periods

The arrhythmic properties outlined above were then compared with the observed differences in electrophysiological, anatomical and molecular properties. The latter were in turn correlated with independent or interacting effects from the *Scn5a*+/− or WT genotype, or recording from the RVOT or RV. Table 1 summarizes the detailed results of this analysis. VERPs could be obtained from PES runs that culminated in refractoriness as opposed to arrhythmia. The *Scn5a*+/− genotype exerted an independent effect of increasing VERP regardless of recording site (Table 1). VERPs were greater in the RVOTs of the *Scn5a*+/− than in the corresponding WT (WT: *n* = 17, 42.65 ± 2.60 ms; *Scn5a*+/− *n* = 5, 70.6 ± 6.24 ms; *P* < 0.01). They were indistinguishable between the RVs of *Scn5a*+/− and WT hearts (*P* > 0.05; WT, *n* = 17, 43.36 ± 4.07 ms; *Scn5a*+/−, *n* = 5, 54.29 ± 6.57 ms).

**Table 1:** Results of two-way anova testing for independent or interacting effects of genotype and anatomical region on electrophysiological properties, Na_v_1.5 expression and fibrotic properties*

	Effect of genotype (*Scn5a*+/− vs. WT)	Effect of anatomical region (RVOT vs. RV)	Presence/absence of interaction	Number (*n*) of *Scn5a*+/− hearts	Number (*n*) of WT hearts
VERP	*P* < 0.001 (*Scn5a*+/− >WT)	*P* > 0.05 (NS)	*P* > 0.05 (NS)	5	17
Conduction velocity from planar fit to LATs (CV†)					
AVN pacing site	*P* < 0.01 (*Scn5a*+/− <WT)	*P* < 0.01	*P* > 0.05 (NS)	17	16
LV base pacing site	*P* < 0.01 (*Scn5a*+/− <WT)	*P* < 0.01	*P* > 0.05 (NS)	10	7
LV apex pacing site	*P* < 0.01 (*Scn5a*+/− <WT)	*P* < 0.01	*P* > 0.05 (NS)	11	6
Conduction velocity from local vector analysis of LATs (CV^*^)					
AVN pacing site	*P* < 0.001 (*Scn5a*+/− <WT)	*P* > 0.05 (NS)	*P* > 0.05 (NS)	17	14
LV base pacing site	*P* < 0.0001 (*Scn5a*+/− <WT)	*P* < 0.01 (RVOT > RV)	*P* > 0.05 (NS)	9	7
LV apex pacing site	*P* < 0.001 (*Scn5a*+/− <WT)	*P* > 0.05 (NS)	*P* > 0.05 (NS)	12	6
Conduction dispersion from local vector analysis of LATs (CD)					
AVN pacing site	*P* > 0.05 (NS)	*P* < 0.001 (RVOT > RV)	*P* > 0.05 (NS)	17	14
LV base pacing site	*P* > 0.05 (NS)	*P* > 0.05 (NS)	*P* > 0.05 (NS)	9	7
LV apex pacing site	*P* > 0.05 (NS)	*P* > 0.05 (NS)	*P* > 0.05 (NS)	12	6
Western blot: Na_v_1.5 expression	*P* < 0.01 (*Scn5a*+/− <WT)	*P* > 0.05 (NS)	*P* > 0.05 (NS)	5	5
Levels of fibrosis	*P* > 0.05 (NS)	*P* < 0.001 (RVOT > RV)	*P* > 0.05 (NS)	4	4

AVN, atrioventricular node; RV, right ventricular; RVOT, right ventricular outflow tract; LV, left ventricular; LATs, local activation times.

* Pacing at the RVOT resulted in higher incidences of arrhythmia in *Scn5a*+/− than WT hearts (Fisher's exact test: *P* < 0.01); Pacing at the RV yielded indistinguishable incidences between WT and *Scn5a*+/−.

### Effective conduction velocities

**Figure 3:**
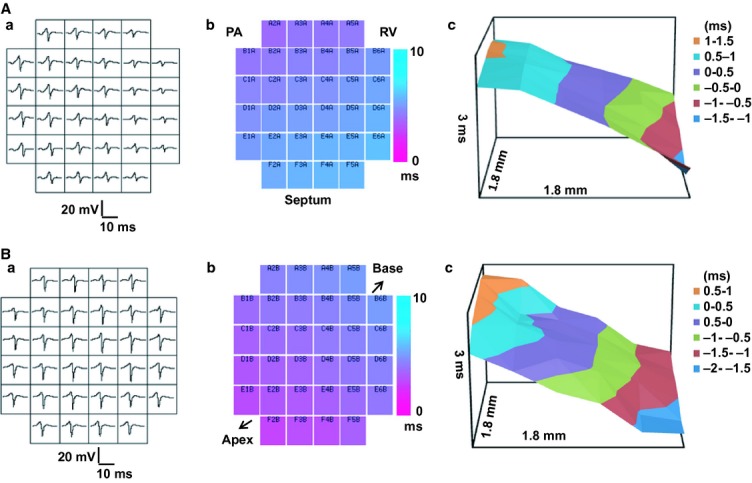
(a, b) Typical multi-electrode array (MEA) recordings from the free walls of the right ventricular outflow tract (RVOT) and right ventricle (RV) in WT hearts. Comparison of typical results obtained from (A) the RVOT, and (B) the RV, including (a) typical traces obtained at each array channel, (b), false colour maps representing their times to first peak, with the rows and columns corresponding to each electrode indicated at the top of each false colour) and (c) plots of local activation times (LATs) (vertical axis) at different positions in the array (*x* and *z* horizontal axes). PA, pulmonary artery; RV, right ventricle.

**Figure 4:**
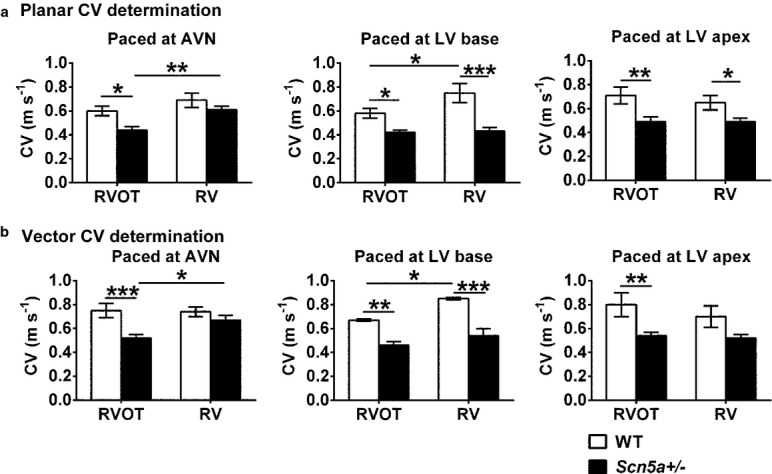
Determinations of effective conduction velocities (CV†) (a) and conduction velocity magnitudes (CV*) (b) from LAT values obtained by multi-electrode array (MEA) recordings at the right ventricular outflow tract (RVOT) (left pair of histograms) and right ventricle (RV) (right pair of histograms) for WT (clear bars) and *Scn5a*+/− hearts (filled bars) during pacing at the atrioventricular node (AVN), LV base and LV apex pacing sites. Pairwise testing in (a) demonstrated that CV† in the RVOT of the *Scn5a*+/− was reduced compared with that of the RVOT of WT with all three pacing sites (*P* < 0.05, 0.05 and 0.01 respectively). In addition, with pacing at the AVN and the LV base, the RVOT showed reduced CV†s compared with the RV in the *Scn5a*+/− (*P* < 0.01) and the WT respectively (*P* < 0.05). Finally, with pacing at the LV base and apex, CV† was reduced in the RV of the *Scn5a*+/− relative to that in the RV of the WT (*P* < 0.0001 and <0.05 respectively). In (b), pairwise testing demonstrated a reduced CV* in the RVOT compared with the RV in the *Scn5a*+/− (*P* < 0.05) with AVN and in the WT (*P* < 0.05) with LV base pacing. CV* was lower in the RVOT of the *Scn5a*+/− than the RVOT of the WT with AVN and LV base and LV apex pacing (*P* < 0.001, <0.01, <0.01 respectively). Finally, there was a reduced CV* in the *Scn5a*+/− RV compared with the WT RV with pacing at the LV base (*P* < 0.0001). *P < 0.05; **P < 0.01; ***P < 0.001.

Epicardial MEA measurements comparing ventricular APs in RVOTs and RVs of WT and *Scn5a*+/− hearts were then made under regular AVN pacing that would be expected to initiate an excitation sequence approximating that expected *in vivo*. Results were compared with findings from stimulation at the LV base and apex. Figure 3A,B exemplifies MEA measurements at the RVOT (A) and RV of WT (B) hearts during regular AVN pacing. It shows (a) traces obtained from each array element and (b) false colour maps representing their times to first peak. (c) The resulting plots of LATs (vertical axis) at different positions in the array (x and z horizontal axes) typically showed smooth surfaces suggesting a graded activation of the epicardial surface. It was therefore possible to derive an effective conduction velocity, CV†, by a least-squares fit of a regression plane through this array of LATs. Figure 4a summarizes the results of these CV† determinations (means ± SEMs) from runs obtained from the RVOTs and RVs of WT and *Scn5a*+/− hearts under each pacing condition.

With pacing at the AVN, LV base and LV apex, both the *Scn5a*+/− genotype and the choice of RVOT as recording site independently produced reductions in CV† (see Table 1 for results of two-way anova). Pairwise testing then demonstrated that the CV† in the RVOT of the *Scn5a*+/− was reduced compared to that of the RVOT of WT. In addition, with pacing at the AVN and the LV base, the RVOT showed reduced CV†s compared with the RV in the *Scn5a*+/− and the WT respectively. Finally, with pacing at the LV base and apex, CV† was reduced in the RV of the *Scn5a*+/− relative to that in the RV of the WT (Fig. 4a; see legend for detailed results of pairwise testing).

### Conduction velocity magnitudes and directions from local vector analysis

The effective conduction velocities determined above are the consequence of both the magnitude, and the direction, *θ*, of AP conduction through the MEA. These component variables were calculated for successive square arrays of four MEA recording sites (see Methods). The magnitude determinations were then averaged over all the determinations made for the array to provide a conduction velocity magnitude, CV*, whose means ± SEMs are shown in Figure 4b. The *Scn5a*+/− genotype resulted in a reduced CV* whether hearts were paced at the AVN, LV base or LV apex. In contrast, choice of RVOT or RV recording site exerted no influence on CV* except with pacing at the LV base (See Table 1 for results of the two-way anova). Pairwise testing demonstrated that CV* was reduced in the RVOT compared with the RV in the *Scn5a*+/− with pacing at the AVN and in the WT with pacing at the LV base. CV* was also lower in the RVOT of the *Scn5a*+/− than the RVOT of the WT with AVN, LV base and LV apex pacing. Finally, there was a reduced CV* in the *Scn5a*+/− RV compared with the WT RV (Fig. 4b; see legend for detailed results of pairwise testing).

**Figure 5:**
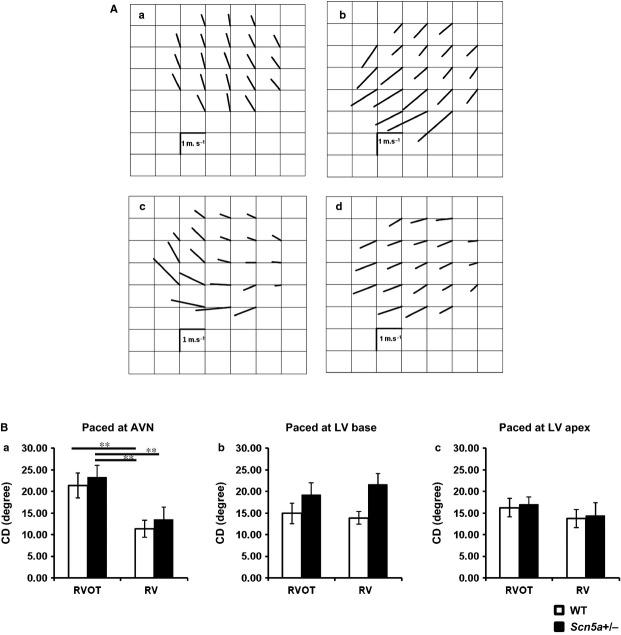
(A) Mappings of conduction directions from recordings made at the free walls of the right ventricular outflow tract (RVOT) and right ventricle (RV) of WT and *Scn5a*+/− hearts. Typical findings from WT (a, b) and *Scn5a*+/− (c, d) with recording from RVOT (a, c) and RV (b, d). (B) Determinations of the dispersion (CD) in conduction direction at the RVOT (left pair of histograms) and RV (right pair of histograms) for both WT (clear bars) and *Scn5a*+/− hearts (filled bars) sorted by the atrioventricular node (AVN) (a), LV base (b) and LV apex (c) pacing sites. CD: dispersion in conduction direction. With AVN pacing (Ba), the *Scn5a*+/− RVOT showed greater dispersions than either the RV of the WT (*P* < 0.01) or the RV of the *Scn5a*+/− (*P* < 0.01). Similarly, the WT RVOT showed greater dispersions than the WT RV (*P* < 0.01) (Ba). **P < 0.01.

It was also possible to obtain estimates of the dispersion in CD, through the MEA recording sites. Figure 5A exemplifies velocity vector maps for the RVOT (Fig. 5Aa,c) and RV (b, d) of WT (a, b) and *Scn5a*+/− (c, d) hearts. Each vector represents the magnitude and direction, *θ*, for each velocity at different positions within the MEA. The dispersion of CD, was then determined from the standard deviations of the values of *θ* obtained through the MEA (means ± SEMs). CD was greater at the RVOT as opposed to RV, with pacing at the AVN, but not at either the LV base or apex. There was no influence from or interaction with genotype (see Table 1 for results of two-way anova). Pairwise testing revealed that with AVN pacing, the *Scn5a*+/− RVOT showed greater dispersions than either the RV of the WT or the RV of the *Scn5a*+/−. Similarly, the WT RVOT showed greater dispersions than the WT RV (Fig. 5Ba; see legend for detailed results of pairwise testing).

### Na_v_1.5 expression and histological properties

**Figure 6:**
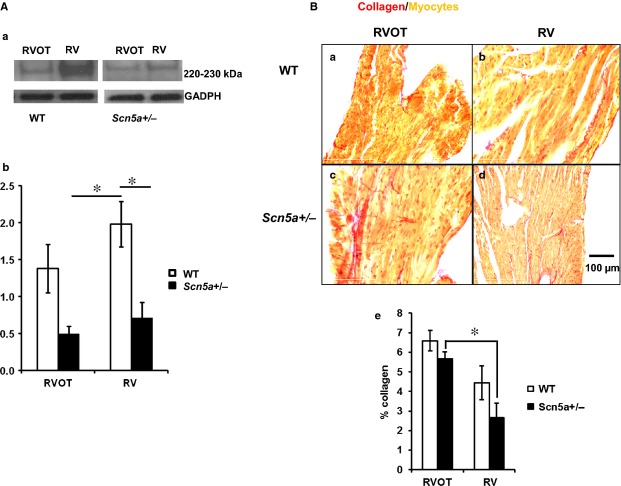
Assessments of Na_v_1.5 expression (A) showing western blot results (a) and their quantification (b) and fibrosis (B) showing typical histological results for WT (a, b) and *Scn5a*+/− (c, d) from the right ventricular outflow tract (RVOT) (a, c) and right ventricle (RV) (b, d), with their quantification (Be). Pairwise testing demonstrated that Na_v_1.5 expression levels (Ab) in both the RV of the *Scn5a*+/− (*P* < 0.05), and the RVOT of the *Scn5a*+/− were lower than in the RV of the WT (*P* < 0.05). It also demonstrated a greater fibrosis, assessed by picrosirius red staining, in the RVOT than in the RV free wall in *Scn5a*+/− hearts (*P* < 0.05) (Be). **P < 0.05.

The final experiments related the above physiological results to biochemical measures of Na_v_1.5 expression and histological measures of fibrotic change in the RVOT and RV of WT and *Scn5a*+/− hearts [Fig. 6Aa,B(a-d)]. Na_v_1.5 expression levels were reduced in association with *Scn5a*+/− genotype independent of whether these were measured in the RVOT or RV (see Table 1 for results of two-way anova). Pairwise tests showed that Na_v_1.5 expression levels (Fig. 6Ab) in both the RV of the *Scn5a*+/−, and the RVOT of the *Scn5a*+/− were lower than in the RV of the WT. In contrast, differences in picrosirius red staining (Fig. 6Ba–d) demonstrated a greater level of fibrosis in the RVOT than the RV free wall regardless of genotype (see Table 1 for results of two-way anova). Pairwise tests then demonstrated greater fibrosis in the RVOT than in the RV free wall in *Scn5a*+/− hearts (Fig. 6Be, see figure legend for detailed results of analysis).

## Discussion

The BrS is an arrhythmic condition clinically characterized by the occurrence of potentially fatal arrhythmic episodes beyond middle age. Up to 30% of BrS patients have *SCN5A* mutations involving Na_v_1.5 (Chen *et al*. 1998, Antzelevitch *et al*. 2007, London *et al*. 2007). Patients with BrS were originally considered to have normal cardiac structure and function, but several studies report progressive myocardial structural abnormalities (Bezzina *et al*. 1999, 2003, Coronel *et al*. 2005, Remme *et al*. 2008, Persson & Persson 2012). Clinical evidence additionally implicates specific anatomical regions, particularly the RVOT, as the site for initiation of arrhythmias with the resulting regional differences in epicardial conduction velocity between the RVOT and RV then triggering epicardial re-entrant excitation waves (Meregalli *et al*. 2005).

Genetically modified mice have proven useful models for observed changes in cardiac arrhythmic disease, particularly BrS (Martin *et al*. 2012, Speerschneider & Thomsen 2013). Both *Scn5a*-1798insD (Stein *et al*. 2009) and *Scn5a*+/− mice carrying loss of function *Scn5a* mutations (Papadatos *et al*. 2002) show conduction defects. They also show a decreased Na^+^ channel function as well as progressive fibrotic invasion between the cardiomyoctes and altered gap junctions (van Veen *et al*. 2005, Stein *et al*. 2009). Both conduction and histological abnormalities were more prominent in the right than the left side of the heart in parallel with the arrhythmic substrate in BrS (Coronel *et al*. 2005, Postema *et al*. 2008, Jeevaratnam *et al*. 2010, 2012, Lodder & Bezzina 2013). However, the related clinical, molecular and biophysical information related to these changes, including the extent to which these changes are involved in disease progression and arrhythmogenesis remain limited (Remme *et al*. 2008).

The present investigations use the *Scn5a*+/− model to explore for the existence and contributions of such factors to its arrhythmic properties. Previous studies demonstrated that the *Scn5a+/*− model (Papadatos *et al*. 2002) reproduces atrial and ventricular arrhythmic (Lei *et al*. 2005, Stokoe *et al*. 2007, Hao *et al*. 2011), electrophysiological (Martin *et al*. 2012), pharmacological (Martin *et al*. 2010a,b) and age-dependent fibrotic, properties (Coronel *et al*. 2005, van Veen *et al*. 2005, Jeevaratnam *et al*. 2010, 2012) associated with human BrS. The present experiments demonstrated and explored RVOT involvement in the arrhythmogenesis observed in isolated perfused whole hearts. They explored for correlations between anatomical regions and genotype, and arrhythmogenesis. They then further correlated these with conduction differences assessed by MEA recording, Na_v_1.5 expression and regional fibrotic change. Findings concerning each of these parameters were then statistically assessed using two-way anova for the presence or absence of either independent or interacting effects of the *Scn5a*+/− vs. the WT genotype, and the RVOT or RV site at which the parameter was measured.

The electrophysiological experiments demonstrated (1) increased arrhythmic tendency with RVOT as opposed to RV stimulation in *Scn5a*+/−, but not WT hearts. This recapitulated clinical studies similarly implicating the RVOT in ventricular arrhythmia in BrS (Morita *et al*. 2003), and previous reports that arrhythmic tendency varies with pacing site (Osadchii 2012). However, (2) VERPs were increased with the *Scn5a*+/− genotype regardless of RVOT or RV recording site. VERPs were greater in the RVOTs of *Scn5a*+/− than in corresponding WT but were indistinguishable between *Scn5a*+/− and WT RVs. These findings contrast with expectations of reduced or unchanged VERPs, predicted by hypotheses invoking Phase II re-entry phenomena arising from altered relative magnitudes of *I*_Na_ and *I*_to_, in arrhythmia in BrS (Lukas & Antzelevitch 1996, Antzelevitch 2006, Veeraraghavan *et al*. 2013). Under conditions of constant velocity, increased VERP would also tend to increase AP wavelength, *λ*, classically defined as the product *λ* = CV† VERP in the *Scn5a*+/− whose arrhythmic substrate would normally be expected to accompany a decreased *λ*. In contrast, (3) both the *Scn5a*+/− genotype and recording from the RVOT as opposed to RV independently resulted in reduced effective CV† determined by a planar fit to LAT results, whatever, the AVN, LV base or LV apex, pacing site. CV† was slowest in *Scn5a*+/− RVOT compared with the remaining *Scn5a*+/− and WT, RV and RVOT recording sites. CV† in turn is determined by (4) the conduction velocity magnitude, CV* and (5) the dispersion in its direction CD, derived from local vector analysis. CV* was reduced with the *Scn5a*+/− as opposed to the WT genotype independently of the choice of RVOT as opposed to RV recording site with AVN pacing. In contrast, CD was increased by the choice of the RVOT as opposed to the RV as recording site, with no influence of or interaction with genotype, with AVN pacing. Further, biochemical and anatomical, studies demonstrated (6) a reduced Na_v_1.5 expression in both RVOT and RV of *Scn5a*+/− attributable solely to genotype and independent of anatomical region. In contrast, (7) interstitial fibrotic changes were greater in the RVOT than the RV in the *Scn5a*+/− but not WT, attributable to anatomical regions independent of genotype.

The results (1)–(7) together demonstrate and explain the highest incidences of arrhythmia in the *Scn5a*+/− RVOT. Admittedly, *Scn5a*+/− resulted in higher VERPs, nevertheless consistent with a reduced Na_v_1.5 expression, that would tend to increase AP wavelength, *λ*, contrary to expectations of arrhythmic substrate. Nevertheless, the observed differences in arrhythmogenicity, correlated with APs in the *Scn5a+/*− RVOT having the lowest CV† and CV*, and the highest CD amongst RV and RVOT recording sites in *Scn5a*+/− and WT hearts. Furthermore, the reductions in CV† reflect both the *Scn5a*+/− genotype, and the choice of RVOT as opposed to RV as a recording site. Of the component variables underlying CV†, reductions in CV* are attributable to the reduced Na_v_1.5 occurring in the *Scn5a*+/− genotype whereas the greater CD reflects the greater fibrosis in the RVOT compared with the RV.

These findings provide a physiological basis for clinical reports describing structural abnormalities and degenerative changes in patients with a *SCN5A* mutation (Bezzina *et al*. 2003). For example, a boy with compound heterozygosity for two *SCN5A* mutations exhibited severe degenerative changes in the specialized conduction system (Bezzina *et al*. 2003). Fatty replacement and fibrosis in the RVOT have been described in patients with BrS despite normal LV anatomy (Thiene *et al*. 1988, Martini *et al*. 1989, Corrado *et al*. 1996). This often involved the RVOT free wall (Tada *et al*. 1998) at which VT or VF was most readily inducible during electrophysiological assessment (Carlson *et al*. 1994, Morita *et al*. 2003, Papavassiliu *et al*. 2004). They also extend recent reports of increased fibrosis with age particularly in male *Scn5a*+/− patients, although the latter studies did not distinguish between RV and RVOT fibrosis (Jeevaratnam *et al*. 2011, 2012).

Together, these results unify a number of clinical observations hitherto not fully studied using experimental models. They demonstrate a greater arrhythmic tendency in the RVOT of the *Scn5a*+/−, attributing this to a combination of reduced Na_v_1.5 expression and increased fibrosis. The increased arrhythmogenesis correlated with *both* (1) a reduced effective CV† associated with *both* reduced Na_v_1.5 expression and increased fibrosis in the RVOT. This in turn reflected (2) a reduced conduction velocity magnitude (CV*) associated only with reduced Na_v_1.5 expression and not increased fibrosis in the RVOT together with (3) an increased CD associated only with increased fibrosis in the RVOT and not reduced Na_v_1.5 expression. Both (2) and (3) combined in the case of the *Scn5a*+/− RVOT would thus increase its arrhythmic tendency in this analysis, that may be applicable to other arrhythmic conditions associated with both channel and structural abnormality (Duehmke *et al*. 2012, Heijman *et al*. 2013).
